# A Genetic Investigation of the KEOPS Complex in Halophilic Archaea

**DOI:** 10.1371/journal.pone.0043013

**Published:** 2012-08-23

**Authors:** Adit Naor, Patrick C. Thiaville, Neta Altman-Price, Ifat Cohen-Or, Thorsten Allers, Valérie de Crécy-Lagard, Uri Gophna

**Affiliations:** 1 Department of Molecular Microbiology and Biotechnology, George S. Wise Faculty of Life Sciences, Tel Aviv University, Tel Aviv, Israel; 2 Department of Microbiology and Cell Science, University of Florida, Gainesville, Florida, United States of America; 3 School of Biology, University of Nottingham, Queen's Medical Centre, Nottingham, United Kingdom; Tulane University Health Sciences Center, United States of America

## Abstract

KEOPS is an important cellular complex conserved in Eukarya, with some subunits conserved in Archaea and Bacteria. This complex was recently found to play an essential role in formation of the tRNA modification threonylcarbamoyladenosine (t^6^A), and was previously associated with telomere length maintenance and transcription. KEOPS subunits are conserved in Archaea, especially in the Euryarchaea, where they had been studied *in vitro*. Here we attempted to delete the genes encoding the four conserved subunits of the KEOPS complex in the euryarchaeote *Haloferax volcanii* and study their phenotypes *in vivo*. The fused *kae1*-*bud32* gene was shown to be essential as was *cgi121*, which is dispensable in yeast. In contrast, *pcc1* (encoding the putative dimerizing unit of KEOPS) was not essential in *H. volcanii*. Deletion of *pcc1* led to pleiotropic phenotypes, including decreased growth rate, reduced levels of t^6^A modification, and elevated levels of intra-cellular glycation products.

## Introduction

Kae1 (also referred to as Gcp or YgjD, and recently renamed TsaD [1]) is one of about 60 proteins conserved in over 99% of organisms with sequenced genomes. Despite this impressive ubiquity, its function has remained elusive until recently, and is yet not fully understood. Kae1 therefore remains a top priority target for experimental study [2]. This protein is part of a complex, named KEOPS or EKC in Eukarya and Archaea [3], [4], [5]. The KEOPS (Kinase, Endopeptidase and Other Proteins of small Size) complex was first characterized in yeast and shown to be involved in the elongation and uncapping of telomeres, as well as a transcription factor. More recently, it was shown to be involved in the synthesis of the universal tRNA modification threonylcarbamoyladenosine or (t^6^A) [6], [7]. The KEOPS complex is composed of Kae1; the serine/threonine kinase-like Bud32; Cgi121; Pcc1, and Gon7 (unique to fungi) [5]. Kae1 is essential in Gram-positive and Gram-negative bacteria [8], [9], [10], in yeast, *kae1*- deficient mutants are very sick [4], [5], [6], [7], [11]. *Saccharomyces cerevisiae* strains carrying deletions of any of the other KEOPS subunit- encoding genes are viable, albeit with reduced growth rates [5], [12].

The bacterial Kae1 homologue TsaD, initially recognized as a secreted glycopeptidase in *Mannheimia haemolytica* [13], was later shown to be involved in the metabolism of Advanced Glycated End Products (AGEs). Depletion of TsaD resulted in cellular accumulation of AGEs followed by growth arrest in *Escherichia coli* cells [10]. AGEs are highly stable, toxic compounds which were found to be involved in several aspects of cell physiology. AGEs accumulate in bacteria and are actively secreted into the growth medium [14]. Several additional phenotypes resulting from TsaD depletion in *E. coli* include deficiencies in DNA maintenance, membrane and cell shape homeostasis, and cell division [15], [16], [17]. Although at least some of these pleiotropic phenotypes could be linked to glycation, a simpler explanation was offered by recent studies that link Kae1/TsaD to the synthesis of t^6^A. This universal modification is found at position 37, 3′ of the anti-codon, in all tRNAs that pair with ANN codons [6], [7]. tRNA extracted from yeast Δ*kae1* mutants lack t^6^A [6], [7], [18], and the TsaD protein from *E. coli,* in combination with TsaB (YrdC), TsaC (YjeE), and TsaE (YeaZ) is required to synthesize t^6^A *in vitro* [1]. As the absence of t^6^A leads to mis-initiation and frameshifts [7], [19], the phenotypes observed could be indirectly linked due to mistranslation of specific proteins, in accordance with the absence of other modifications of the anticodon such as cm^5^U derivatives in yeast [20], [21]. The eukaryotic and bacterial t^6^A machinery must have diverged as both TsaD/Kae1 and YrdC/Sua5 are universal, but TsaE and TsaB are bacterial-specific. Sua5 and Kae1 alone are not sufficient to synthesize t^6^A in the cytosol [6]. One possibility is that the other subunits of the KEOPS complex found in Eukaryotes and Archaea are required, as it was previously shown that mutations in *pcc1* and *bud32* in yeast affect t^6^A levels [6], [22].

Although archaeal proteins served as a structural model for the eukaryotic KEOPS, no *in vivo* studies of the KEOPS complex in Archaea have been reported. Previous studies on KEOPS combined *in vitro* analysis of archaeal proteins with *in vivo* genetic analysis in yeast, and were useful for determining some of the functional roles of the eukaryotic KEOPS. An *in vitro* study of the Kae1 homolog of the hyperthermophilic archaeon *Pyrococcus abyssi* showed it to be an endonuclease of apurinated nucleotides [23], hinting that it may be involved in DNA repair stability or processes. However, a similar study of the *Methanococcus jannaschii* homolog concluded that the latter Kae1 homolog does not possess such activity and cannot bind DNA [4]. Reconstruction and structural analysis of the entire KEOPS complex using proteins from *Methanococcus jannaschii* and *Pyrococcus furiosus* expressed in *E. coli*, suggested that Cgi121 activates Bud32 which in turn regulates Kae1 activity, while Pcc1 is a dimerizing module [4].

However, since *in vivo* experiments have not been carried out in Archaea, and attempts to complement the yeast or bacterial *kae1/tsaD* deletions with archaeal homologs failed [7], no physiological role could be assigned to the archaeal KEOPS. Here we perform a genetic analysis in the model archaeon *Haloferax volcanii* and establish the essentiality of this complex in the third domain of life.

## Results

### The Kae1-Bud32 fusion protein is essential in *Haloferax volcanii*

Four components of the KEOPS complex– Kae1, Bud32, Cgi121 and Pcc1 have homologs in Archaea [4]. Homologs of these four components were identified in the genome of *H. volcanii* using BLASTP [24]. The genes encoding Kae1 and Bud32 are fused to a single open reading frame in haloarchaea and methanogens [23].

To determine the essentiality of the Kae1-Bud32 fusion encoding gene in *H. volcanii* (gene number HVO_1895), we employed the “pop-in/pop-out” strategy for gene deletion [25], [26] see materials and methods. We attempted to delete the *kae1*-*bud32* gene, using plasmid pAN1 transformed into *H. volcanii* strain H26 creating the pop-in strain H-AN2 (For plasmids and primers used see tables S1 and S2, respectively). Following counter selection (“pop-out”), 30 colonies were screened, but no *kae1*-*bud32* deletions were obtained, suggesting that this fusion gene is probably essential. We then proceeded to perform a gene replacement experiment, replacing *kae1*-*bud32* with a *trpA* selectable marker (using plasmid pAN2). pAN2 was transformed into *H. volcanii* strain H133 (Δ*pyrE*, Δ*trpA*), creating the pop-in strain H-AN1. Following counter selection (“pop-out”), no colonies were obtained, supporting the essentiality of *kae1-bud32* in *H. volcanii*. To validate this conclusion, a plasmid carrying the complete *kae1-bud32* gene was cloned into the pRV1-Ptna-*bgaH* plasmid [27] placing *kae1-bud32* under the control of the tryptophanase promoter (pAN4). This plasmid was subsequently transformed into the “pop-in” strain, HAN2. In this genetic background, where an exogenous gene is provided, it was possible to knock out the chromosomal gene and obtain strain HAN4 (Figure S1A).

A similar approach was used to examine the essentiality of both components of the fusion gene, *kae1* and *bud32*. Fragments encoding each of the putative functional domains were cloned into the pRV plasmid and transformed into strain HAN1. The fusion gene was split to contain the individual domains, as previously done for the *Methanocaldococcus jannaschii* homolog [4] (for the exact positions of the fragment inserted, see table S1). If only one of the genes/domains is essential, one should be able to replace the fusion gene region with a *trpA* selectable marker, provided that the other portion is supplied exogenously (using plasmids pAN 12 and pAN 19). However, this was also unsuccessful, showing that both the Kae1 and Bud32 domains are likely to be essential.

### Cgi121 is essential in *Haloferax volcanii*

The “pop-in/pop-out” methodology described above was also used in an attempt to delete *cgi121* (gene number HVO_0013) using plasmid pAN23. When attempting to delete *cgi121* with no selectable marker, 50 “pop-out” colonies were examined, and were found to have reverted back to wild-type. Moreover, when attempting to replace *cgi121* with the *trpA* selectable marker (pAN24), as described above for *kae1-bud32,* it proved impossible to delete *cgi121*. When *cgi121* was provided on a complementing plasmid, pAN25, we were able to delete *cgi121* from its chromosomal location. This verifies that failure to knock out the gene is due to its essentiality and not a technical artifact (Figure S1B).

### A *Haloferax volcanii* Δ*pcc1* mutant displays reduced growth

**Figure 1:**
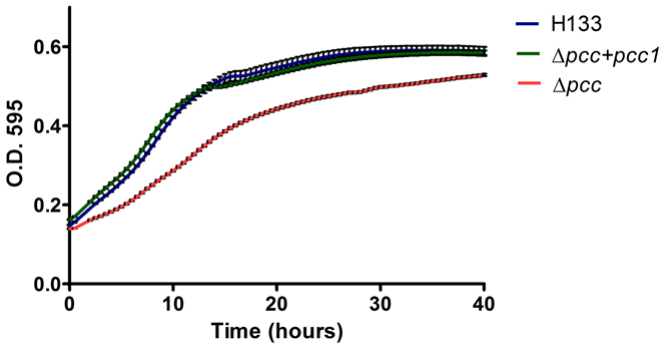
Growth of wild type *Haloferax volcanii* and the Δ*pcc1* mutant cells. Growth curves comparing the growth rate of H133 (wild-type), Δ*pcc1* (HAN16) and Δ*pcc1* containing the complementing plasmid (HAN19). Results are a mean of 10 replicates, error bars indicates standard error of the mean.

Our attempts to delete *pcc1* (HVO_0652) by “pop-in/pop-out” (using plasmid pAN21) without a selectable marker were unsuccessful, indicating much reduced fitness. The 50 “pop-out” colonies that were scanned were found to have reverted back to the wild-type genotype. However, when replacing the gene with the *trpA* selectable marker in an H133 (*trp* ^−^) background (using plasmid pAN22), some colonies were obtained following counter-selection. Since the “pop-out” colonies were smaller in size (Figure S1C). We therefore tested the growth rate of Δ*pcc1.* As seen in Figure 1, the growth rate of Δ*pcc1* was significantly lower when compared to the H133 (wild-type) strain. To confirm that this significant decrease in growth rate is not due to other factors, we introduced a complementing plasmid (pAN26) containing *pcc1 in trans*. The growth rate of the complemented strain is similar to that of the wild type, indicating that the loss of *pcc1* causes a reduced growth rate.

### A *Haloferax volcanii* Δ*pcc1* mutant contains more AGEs

**Figure 2:**
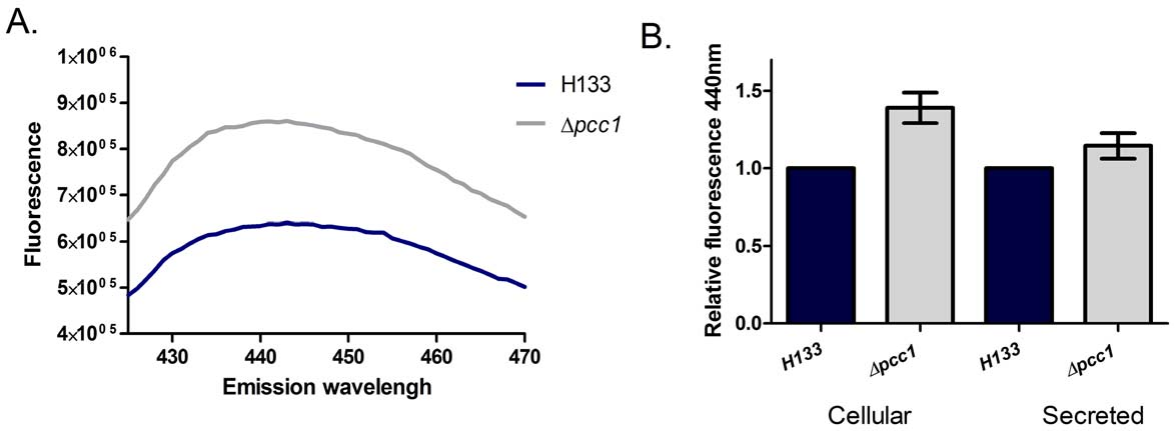
Advanced glycation products in wild type *Haloferax volcanii* and the Δ*pcc1* mutant cells. **A**. Measurement of AGEs-specific fluorescence in the H133 (wild-type) and in Δ*pcc1* (HAN16). The emission spectrum from 400 nm to 480 nm upon excitation at 370 nm is presented. A representative sample is shown. **B**. Accumulation and secretion of AGEs. AGE-specific fluorescence is shown relative to the wild-type. The results represent three independent experiments, error bars indicates standard error of mean.

Since the bacterial homolog of Kae1 was shown to be involved in the accumulation of cellular AGEs, [10] we compared the levels of AGEs in wild-type *H. volcanii* cells to HAN16 Δ*pcc1* cells. The presence of AGEs can be determined by AGE-specific fluorescence, measured by emission at 440 nm upon excitation at 370 nm (see materials and methods). As shown in Figure 2A, lysates of wild-type cells (H133) showed a flattened peak at about 440 nm, indicating that AGEs were formed. Comparing the level of cellular AGEs in Δ*pcc1* cells to wild-type, we observed a 37% increase of cellular levels of AGEs (Figure 2A). The differences in the level of secreted AGEs were more subtle - about 14% more AGEs were secreted in the Δ*pcc1* cells. Thus, *pcc1*-null cells contain more AGEs, and AGEs secretion probably cannot keep up with the increased accumulation of AGEs.

### A *Haloferax volcanii* Δ*pcc1* mutant has higher nucleic acid content

**Figure 3:**
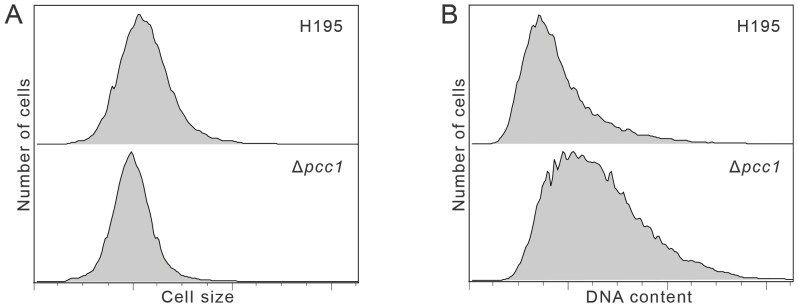
Flow cytometry analysis of *Haloferax volcanii* H195 and H133 Δ*pcc1* mutant cells. H195 (based on H133, with a *bga*Ha-Bb deletion, and a *leuB*- *Ag1* allele, which has flow cytometry profiles highly similar to H133– data not shown) **A.** Cell size as determined by forward light scatter of H195, and of Δ*pcc1* (HAN16). **B.** Nucleic acid content as determined by acridine orange fluorescence of H195 (wild-type) and in Δ*pcc1* (HAN16).

In order to assess the nucleic acid content and distribution in the Δ*pcc1* cells, we examined the cells in a flow cytometer (see materials and methods). The cell sizes of wild-type and Δ*pcc1* were similar (Figure 3A). Cell morphology was also similar under light microscopy (data not shown). Although cell size was normal, nucleic acid content was substantially higher with a broader distribution across cells in the Δ*pcc1* mutants (Figure 3B).

### A *Haloferax volcanii* Δ*pcc1* mutant contains less t^6^A

**Figure 4:**
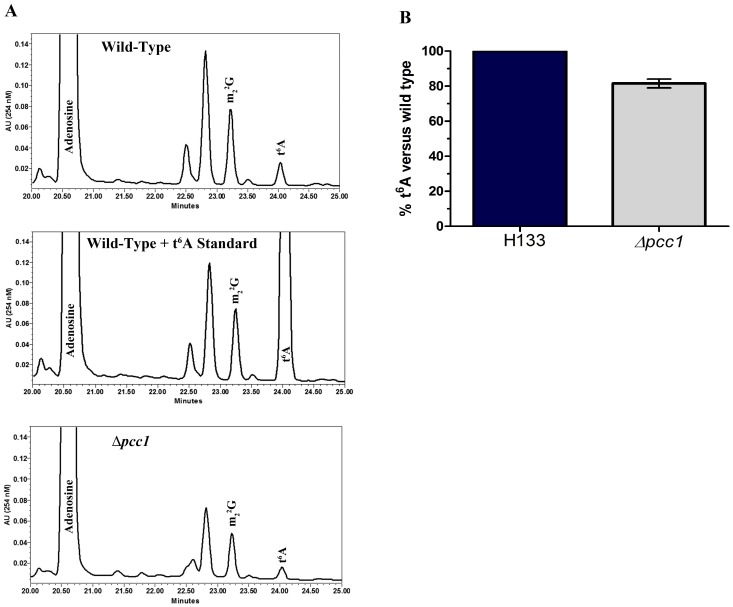
Nucleoside analysis analysis in wild type *Haloferax volcanii* and the Δ*pcc1* mutant cells. tRNAs were extracted from each strain and hydrolyzed to nucleosides. The nucleoside content was determined by HPLC with detection by UV/Vis at 254 nm. Analyses were performed in triplicate from independent cultures. **A**. HPLC chromatographs of nucleosides from wild-type, wild-type with synthesized t^6^A added, or Δ*pcc1*. t^6^A elutes at 24 minutes. **B**. Comparison of the t^6^A peak area of wild-type and Δ*pcc1*. The ratios of Ψ-modified base/m_2_^2^G were used to normalize tRNA concentrations across samples with t^6^A peak area of wild-type set at 100%. Δ*pcc1* contains approximately 19% less t^6^A than wild-type (*P* = 0.02).

tRNAs were extracted from the *Δpcc1* and H133 (wild-type) strains, hydrolyzed to liberated nucleosides, and analyzed by both HPLC and LC/MS/MS. t^6^A was present in Δ*pcc1,* but the mutant strain contained approximately 15–20% less t^6^A relative to H133 (Figure 4). This result is similar to the reduction seen in a *pcc1–4* point mutant in yeast [22]. These results suggest that Pcc1 is not essential for t^6^A formation in Archaea or Eukarya, but contributes to the efficiency of its biosynthesis.

## Discussion

Whether the KEOPS complex is involved in one biological pathway or process that has pleiotropic effects or whether it is directly involved in several pathways is still unclear. Four of the five KEOPS proteins show extraordinary conservation from Archaea to mammals. Notably, one of the proteins, Kae1, is one of the very few proteins encoded by nearly all genomes sequenced in the three domains of life.

This work represents the first *in vivo* investigation of the KEOPS complex in Archaea. We have shown the critical importance of all four KEOPS components for growth of *H. volcanii*. Viability of the *pcc1* deletion mutants was somewhat expected, since Pcc1 is thought to function as a dimerizing module connecting two KEOPS complexes together. Absence of Pcc1 should result in reduced KEOPS activity rather than a complete loss of KEOPS function [4]. Nevertheless, *pcc1* deletion mutants are slow growing, and show several other defects that were previously shown to be associated with disruption of this complex. These phenotypes include an elevated level of AGEs, as was seen in *E. coli* TsaD depletion [10], reduced t^6^A modification, as seen in *E. coli* and *S. cerevisiae*, and an aberrant distribution of nucleic acid content in mutant cells [6], [18]. Curiously, a *cgi121* deletion, which had little effect in yeast, proved to be essential in *H. volcanii*. In yeast, *cgi121* null mutants show no altered t^6^A modification levels [6], perhaps indicating that the KEOPS complex is involved in more than one key cellular functions. This conclusion is further supported by the relative mildness of the t^6^A phenotype of the *pcc1* mutant compared to the substantial growth impairment of that mutant in *H. volcanii*.

The fact that both AGEs-related and t^6^A-related phenotypes were observed in *H. volcanii* raises an interesting question - is the KEOPS complex involved in both protection from glycation damages and the carbamoylation reaction independently, or are these processes linked? One possibility that we are exploring is, that in the absence of a functional KEOPS complex, the TsaC/Sua5 enzyme generates a glycation product. Alternatively, translation errors caused by insufficient t^6^A modification of tRNAs can result in mistranslated proteins that have more exposed lysine residues, providing more abundant substrates for Amadori product formation and results in higher levels of AGEs. Regardless of the exact biochemical cross-talk between Amadori product formation and tRNA t^6^A modification, it is clear that defects in these processes are likely to have pleiotropic phenotypes, such as the differences in nucleic acid content observed in *pcc1*-null mutants.

The question of the essentiality of t^6^A in Archaea remains unanswered. Yeast mutants that lack t^6^A are viable, however *E. coli* mutants are not [6], [7], [28]. Naturally, one wonders whether essentiality is because of the absence of the t^6^A modification itself, or because t^6^A synthesis proteins have additional roles in *E. coli*. The fact that all four *E. coli* t^6^A biosynthesis proteins are essential strongly suggests that it is the modification itself that is essential. Members of the archaeal Sua5 family have been shown to complement both the t^6^A deficiency of the *sua5*Δ yeast mutant and the essentiality phenotype of *E. coli* Δ*tsaC* [28]. Like *E. coli* and unlike yeast, the *H. volcanii* Sua5 homolog HVO_0253 is essential [18]. One possibility explaining the essentiality of t^6^A in prokaryotes and not in eukaryotes is that C34 in tRNA^Ile^_CAU_, decoding AUA codons, has to be modified to lysidine (k^2^C) in Bacteria and agmatidine (agm^2^C) in Archaea to function in decoding [29], [30], [31]. Both the lysidine synthase (TilS) encoding gene and the agmitidine synthase (TiaS) encoding genes are essential [18], [32]. If t^6^A is required for TilS and TiaS activity then t^6^A would be essential in prokaryotes and not in eukaryotes. We are currently investigating this scenario.

## Materials and Methods

### Strains

Strains used in this work and their genotype are listed in Table 1.

**Table 1:** Strains used in this study.

strain	Description	Source
H26	Δ*pyrE2*	[25]
H133	Δ*pyrE2* Δ*trpA* Δ*leuB* Δ*hdrB*	[25]
H195	Δ*pyrE2* Δ*trpA* Δ*hdrB bga*Ha-Bb *leuB*- *Ag1*	[37]
HAN1	H133 with pAN2 pop in.	This work
HAN2	H26 with pAN1 pop in.	This work
HAN4	Deletion of *kae1-bud32* (from pop in strain HAN2) with the pAN4 complementing plasmid	This work
HAN16	Replacement of *pcc1* with a *trpA* cassette (in a H133 background).	This work
HAN19	HAN 16 containing the pAN26 complementing plasmid.	This work

### Culture conditions

*H. volcanii* was routinely grown in rich (HY) medium, or on a selective CA medium (see [33]. For counter-selection of uracil auxotrophs, 5-fluoroorotic acid (5-FOA) (United States Biological) was added to the medium at a final concentration of 100 µg/ml. When required, uracil was added to a final concentration of 50 µg/ml. When needed, novobiocin (Sigma-Aldrich) was added to the medium at a final concentration of 2 µg/ml.

### Transformation

Transformation of *H. volcanii* was carried out using the PEG method as described in [34].

### Gene knockouts

The gene knockouts were performed according to the protocol described in [25], [26]. In this method, the upstream and downstream flanking regions of the sequence to be exchanged are amplified by PCR and cloned together into the ‘suicide plasmid’ pTA131 that carries the *pyrE*2 selectable genetic marker and cannot replicate autonomously in *H. volcanii*. The plasmids are then transformed into a *H. volcanii* Δ*pyrE* mutant, and transformants, in which the plasmids have been integrated into the chromosome, are selected for on plates that lack uracil (‘pop-in’). Upon counter-selection on plates containing uracil and 5-fluoroorotic acid (5FOA), the only cells that survive are those in which the integrated plasmids have been excised by spontaneous intra-chromosomal homologous recombination (‘pop-out’), either restoring the wild-type gene or resulting in allele exchange. Gene replacements were performed as described in [25]. CA medium was used as a uracil- and tryptophan-minus medium for *trpA* cassette selection.

### Growth curves

The growth curves were performed in 96-well plates at 42°C with continuous shaking, using the Biotek ELX808IU-PC microplate reader. Optical density was measured every 30 minutes at a wavelength of 595 nm.

### Flow cytometry

Exponential phase samples (A600∼0.5) were stained with acridine orange (10 µg/ml, Sigma-Aldrich) for two minutes. Flow cytometry was immediately performed using an Apogee A40 equipped with a 50 mW 488 nm solid state laser (Coherent) and a 510–580 nm bandpass filter. Settings LS1: 420 V, FL1: 495 V, 50000 cells. Doublet signals were removed by gating on peak/area plots for LS1 and FL1. Data analyzed using FlowJo (TreeStar Inc).

### Determination of AGEs

Advanced Glycation End-products (AGEs) were quantified using the natural AGE-specific fluorescence (Ex. 370 nm, Em. 440 nm) by scanning emission ranging from 400 nm to 500 nm upon excitation at 370 nm at 37°C, in a HORIBA scientific FluoroLog-3 Spectrofluorometer. Data represent either the full range spectrum, or the 440 nm emission peak, as indicated in the legends. In order to quantify AGEs, the culture was grown to exponential phase (OD_600_∼0.5), and washed once with 18% NaCl solution. When quantifying secreted AGEs, cells were incubated in the salt solution for 3 hours, precipitated by centrifugation and the solution was used for measurement. For the cellular AGEs quantification, cells were washed, lysed by sonication and the lysate was used for measurement.

### Preparation of Bulk tRNA

*H. volcanii* H133 (wild-type) and Δ*pcc1* were grown in YPC [25]. Bulk tRNA was prepared by double phenol/chloroform extraction previously described [35]. Nucleoside preparations were prepared by incubating 100 µg of linearized bulk tRNA with 10 units of Nuclease P1 (Sigma) in 10 mM ammonium acetate (pH 5.3) overnight at 37°C. The next day, the digestion was adjusted to a final concentration of 100 mM ammonium bicarbonate, 0.01 units of Phosphodiesterase I (Sigma), 0.05 units *E. coli* alkaline phosphatase (Sigma), and incubated for an additional 2 hours at 37°C. The hydrolyzed nucleosides were further purified by filtering through a 5 Kd MCO filter (to remove enzymes), dried, and suspended in 20 µL of water prior to analysis by HPLC or LC-MS/MS. All tRNA extractions were performed in triplicate from independent cultures.

### HPLC and LC-MS/MS Analysis

t^6^A was detected by HPLC as described by [36] and also by LC–MS/MS as described in [28]. Levels of t^6^A were measured by integrating the area under the peak from the extraction ion chromatograms. The ratios of Ψ-modified base/m_2_^2^G were used to normalize for tRNA concentrations across samples. t^6^A levels for Δ*pcc1* were compared to wild-type levels, with wild-type set at 100%. The MS/MS fragmentation data as well as a synthesized t^6^A standard provided by D. Davis were used to confirm the presence of t^6^A.

## Supporting Information

Figure S1 Confirmation of genetic mutations. Oligonucleotides anneal outside of the open reading frame, but within the region of homology used for “pop-in/pop-out” deletions. A. PCR analysis of *kae1-bud31* fusion using primers AP26 and AP27. Lane 1 - DNA ladder, lane 2 - wild type. Lane 3- the “pop in” strain (HAN1). Lane 4 – “pop out” of *kae1-bud32* with *kae1-bud32* supplied *in trans* (HAN4). B. PCR analysis of *cgi121* using primers AP229 and AP233. Lane 1- DNA Ladder. Lane 2 – “pop in” of plasmid pAN24 in H133. Lane 3 - DNA Ladder. Lane 4- wild-type. Lane 5 – “pop out” of *cgi121* with *cgi121* supplied *in trans* (pAN25). C. PCR analysis of *pcc1* using primers AP295 and AP296. Lane 1- wild-type. Lane 2 – “pop in” of plasmid pAN22 in H133. Lane 3 and 4 “pop out” HAN16. Lane 5- DNA Ladder.
(TIF)

Table S1 Plasmids used in this study.
(DOCX)

Table S2 Oligonucleotides used in this study.
(DOCX)
