# Supplementary figures and images for: A Genetic Investigation of the KEOPS Complex in Halophilic Archaea
Source: PLoS One. 2012 Aug 23;7(8):e43013. doi: 10.1371/journal.pone.0043013 (PMC3426518; doi:10.1371/journal.pone.0043013)

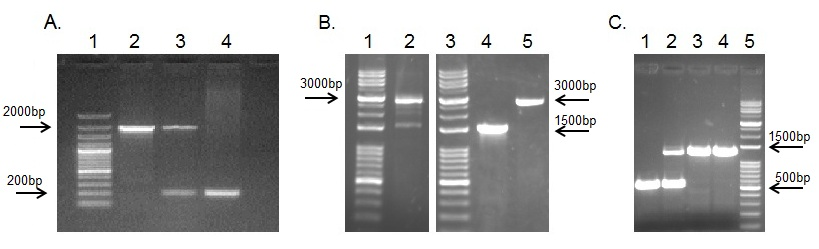

Supplement: Figure S1 — Confirmation of genetic mutations. Oligonucleotides anneal outside of the open reading frame, but within the region of homology used for “pop-in/pop-out” deletions. A. PCR analysis of kae1-bud31 fusion using primers AP26 and AP27. Lane 1 - DNA ladder, lane 2 - wild type. Lane 3- the “pop in” strain (HAN1). Lane 4 – “pop out” of kae1-bud32 with kae1-bud32 supplied in trans (HAN4). B. PCR analysis of cgi121 using primers AP229 and AP233. Lane 1- DNA Ladder. Lane 2 – “pop in” of plasmid pAN24 in H133. Lane 3 - DNA Ladder. Lane 4- wild-type. Lane 5 – “pop out” of cgi121 with cgi121 supplied in trans (pAN25). C. PCR analysis of pcc1 using primers AP295 and AP296. Lane 1- wild-type. Lane 2 – “pop in” of plasmid pAN22 in H133. Lane 3 and 4 “pop out” HAN16. Lane 5- DNA Ladder. (TIF) [file pone.0043013.s001.tif]
