# Supplementary material for: A Genetic Investigation of the KEOPS Complex in Halophilic Archaea
Source: PLoS One. 2012 Aug 23;7(8):e43013. doi: 10.1371/journal.pone.0043013 (PMC3426518; doi:10.1371/journal.pone.0043013)
Supplement: Table S1 — Plasmids used in this study. (DOCX) [file pone.0043013.s002.docx]

**Table S1. Plasmids used in this study.**

| **Plasmid** | **Description** | **Source** |
| --- | --- | --- |
| pTA131 | pBluescript II containing the *H. volcanii* *pyrE2* gene*-* used for 'pop-in' 'pop-out' experiments in' 'pop-out' experiments. | [[1](#_ENREF_1)] |
| pAN1 | pTA131 containing flanking regions for the deletion of the *kae1 bud32* gene. | This work |
| pAN2 | pTA131 containing flanking regions for the deletion of the replacement for *kae1 bud32* gene with *trpA*. | This work |
| pRV | pRV1 containing novobiocin resistance and the Ptna promoter. | [[2](#_ENREF_2)] |
| pAN4 | PRV1-containing the *kae1bud32* gene under the tryptophanase promoter. | This work |
| pAN12 | pRV1 containing only the *bud32* domain under the trptophanase promoter. Contains residues 1-361 of the *kae1 bud32* gene. | This work |
| pAN19 | pRV- containing only the *kae1* domain under the trptophanase promoter. Contains residues 354-551 of the *kae1 bud32* gene. | This work |
| pTA927 | Overexpression vector with pHV2 origin and *pyrE2* marker, for trptophan inducible gene expression. | [[3](#_ENREF_3)] |
| pTA1131 | pTA927 with the *pyrE* replaced with *hdrB* | [[3](#_ENREF_3)] |
| pAN21 | pTA131 containing flanking regions for the deletion of *pcc1*. | This work |
| pAN22 | pTA131 containing flanking regions for the deletion of the replacement for PCC1 gene with *trpA*. | This work |
| pAN23 | pTA131 containing flanking regions for the deletion of the *cgi121* gene. | This work |
| pAN24 | pTA131 containing flanking regions for the deletion of the replacement for *cgi121* gene with *trpA*. | This work |
| pAN25 | pTA1131 with the *cgi121* gene under the tryptophanase promoter. | This work |
| pAN26 | pTA1131 with the *pcc1* gene under the tryptophanase promoter. | This work |

REFERENCES

1. Allers T, Ngo HP, Mevarech M, Lloyd RG (2004) Development of additional selectable markers for the halophilic archaeon Haloferax volcanii based on the leuB and trpA genes. Appl Environ Microbiol 70: 943-953.

2. Large A, Stamme C, Lange C, Duan Z, Allers T, et al. (2007) Characterization of a tightly controlled promoter of the halophilic archaeon *Haloferax volcanii* and its use in the analysis of the essential *cct1* gene. Mol Microbiol 66: 1092-1106.

3. Allers T, Barak S, Liddell S, Wardell K, Mevarech M (2010) Improved strains and plasmid vectors for conditional overexpression of His-tagged proteins in Haloferax volcanii. Appl Environ Microbiol 76: 1759-1769.
