# Supplementary material for: A Genetic Investigation of the KEOPS Complex in Halophilic Archaea
Source: PLoS One. 2012 Aug 23;7(8):e43013. doi: 10.1371/journal.pone.0043013 (PMC3426518; doi:10.1371/journal.pone.0043013)
Supplement: Table S2 — Oligonucleotides used in this study. (DOCX) [file pone.0043013.s003.docx]

**Table S2. Oligonucleotides used in this study.**

| **Name** | **Description** | **Sequence** |
| --- | --- | --- |
| **AP1** | Creation of pAN1 | aagctttcgtcgcgcccatcgac |
| **AP2** | Creation of pAN1 | gaattccgagtgtgaagaaacggcg |
| **AP4** | Creation of pAN1 | gcggccgcggaccttccccgcgttcgt |
| **AP5** | Creation of pAN1 | gaattctaggtcgccgcggactcc |
| **AP3** | Creation of pAN2 (together with AP1,AP2 and AP5) | tctagataggtcgccgcggactcc |
| **AP26** | Examination of *kae1-bud32* deletion | aagttcggcatgcgcaagac |
| **AP27** | Examination of *kae1-bud32* deletion | ggccttccgatggggagtc |
| **AP28** | Creation of pAN4 | catatgcgcattctcggaatcgaagg |
| **AP29** | Creation of pAN4 | gaattctccgcggcgacctaccggta |
| **AP56** | Creation of pAN19 (together with AP29) | acatatg tacaccgacgacggcgcgat |
| **AP84** | Creation of pAN12 (together with AP28) | aagcttctacgcgccgtcgtcggtgt |
| **AP224** | Creation of pAN21 | ttaaagcttagctgttccatcaccgcgagt |
| **AP225** | Creation of pAN21 | aaagaattcggggcgcacgctactcgac |
| **AP226** | Creation of pAN21 | aaatctagaggagtccgcgtaggagaccg |
| **AP228** | Creation of pAN21 | aaagcggccgccgtcgtcggcgtcgtcgg |
| **AP227** | Creation of pAN22 (together with AP224,225,228) | Aaatctagaggagtccgcgtaggagaccg |
| **AP229** | Creation of pAN23 | ttaaagcttggacgaagaccgcatcaccga |
| **AP230** | Creation of pAN23 | aaagaattcgacggccgcgatgaacgaatc |
| **AP231** | Creation of pAN23 | aaagaattcgcggttttcgcggcccgtat |
| **AP233** | Creation of pAN23 | aaagcggccgcttgtaggcgatgtaggggttgt |
| **AP232** | Creation of pAN24 (together with AP229,230,233) | aaatctagagcggttttcgcggcccgtat |
| **AP270** | Creation of pAN26 | CATATGCGCCCCGCGCACAG |
| **AP271** | Creation of pAN26 | AAGCTTCTACGCGGACTCCGACAGCG |
| **AP273** | Creation of pAN25 | CATATGAGGCTCCTCGAAGCCGA |
| **AP274** | Creation of pAN25 | GAATTCTTAGCGGTCGACCGCGAG |
| **AP295** | Examination of *pcc1* deletion | caatgagctacaagtgttcccggtg |
| **AP296** | Examination of *pcc1* deletion | tctcggaggactgcttctgctcgg |
